# Data regarding M1 muscarinic receptor-mediated modulation of hepatic catalase activity in response to oxidative stress

**DOI:** 10.1016/j.dib.2015.12.025

**Published:** 2015-12-18

**Authors:** Ravirajsinh N. Jadeja, Nathalie H. Urrunaga, Daniel Ahmad, Sandeep Khurana

**Affiliations:** a Division of Gastroenterology and Hepatology, Medical College of Georgia, Augusta, GA 30912, USA; b Division of Gastroenterology and Hepatology, University of Maryland School of Medicine, Baltimore, MD 21201, USA; c Department of Medicine, University of Maryland School of Medicine, Baltimore, MD 21201, USA

**Keywords:** Muscarinic receptors, Oxidative stress, Catalase

## Abstract

We recently demonstrated the role of M1 muscarinic receptors (M1R) in modulating oxidative stress in liver and hepatocytes (Urrunaga et al., 2015) [1]. Here we provide data regarding the effect of a novel M1R agonist, VU0357017 (Lebois et al., 2010) [2], on H_2_O_2_-mediated hepatocyte injury, the effect of an M1R antagonist VU0255035 (Sheffler et al., 2009) [3] on catalase and super oxide dismutase (SOD) activities in H_2_O_2_–treated hepatocytes *in vitro*, and finally, the effect of M1R ablation on hepatic catalase activity in acetaminophen (APAP)-treated mice.

**Specifications Table**

**Table:** 

Subject area	*Biology*
More specific subject area	*M1 muscarinic receptors and oxidative stress*
Type of data	*Figures*
How data was acquired	*BioMate 3S Spectrophotometer and Versa Max micro-plate reader*
Data format	*Analyzed*
Experimental factors	*No pretreatment*
Experimental features	*Enzyme activity and cytotoxicity assay*
Data source location	*Medical College of Georgia, Augusta, GA*
Data accessibility	*The data are supplied with this article*

**Value of the data**

In response to oxidative stress:

• In contrast to M1R inhibition that reduces hepatocyte injury [1], M1R activation has no effect.
• In addition to enhancing GSH recovery [1], M1R inhibition enhances hepatic catalase activity.
• However, M1R inhibition has no effect on hepatocyte SOD activity.

## 1 Data

**Fig. 1:**
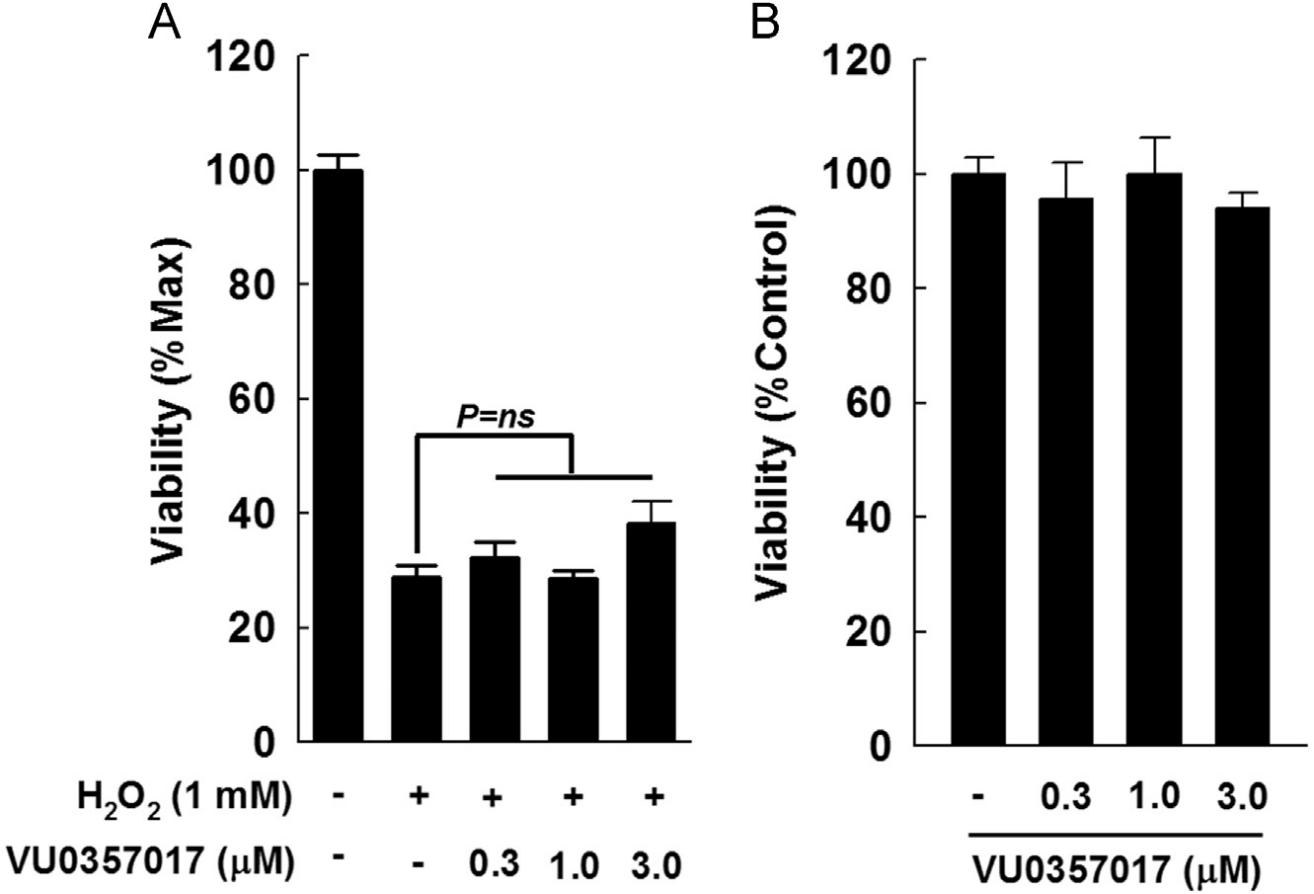
Effect of an M1R agonist (VU0357017) on H_2_O_2_-induced cytotoxicity assessed by MTT assay. (A) Incubation with 1 mM H_2_O_2_ for 6 h markedly reduced AML12 hepatocyte viability. (B) Treatment with VU0357017 (0.3–3.0 µM) alone had no effect on hepatocyte viability. Results are mean±S.E.M. ns: not significant.

**Fig. 2:**
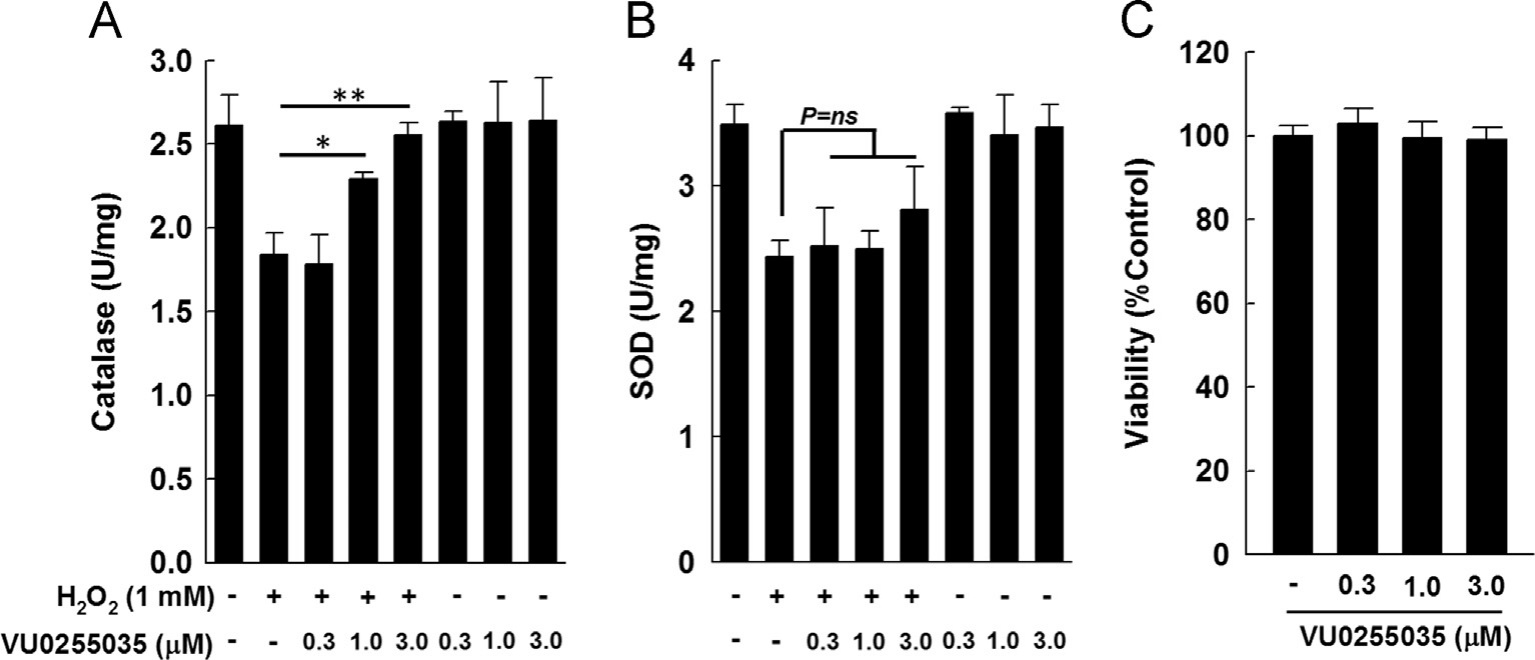
Effects of a highly selective M1R antagonist VU0255035, on catalase and SOD activities were assessed in AML12 hepatocytes treated with 1 mM H_2_O_2_ for 120 min. Compared to cells treated with vehicle (DMSO) alone, catalase and SOD activities were significantly reduced in H_2_O_2_-treated cells. Co-treatment with VU0255035 improved catalase activity (A), but had no effect on SOD activity (B). (C) VU0255035 alone had no effect on cell viability. Results are mean±S.E.M. * *P*<0.05, ** *P*<0.01.

**Fig. 3:**
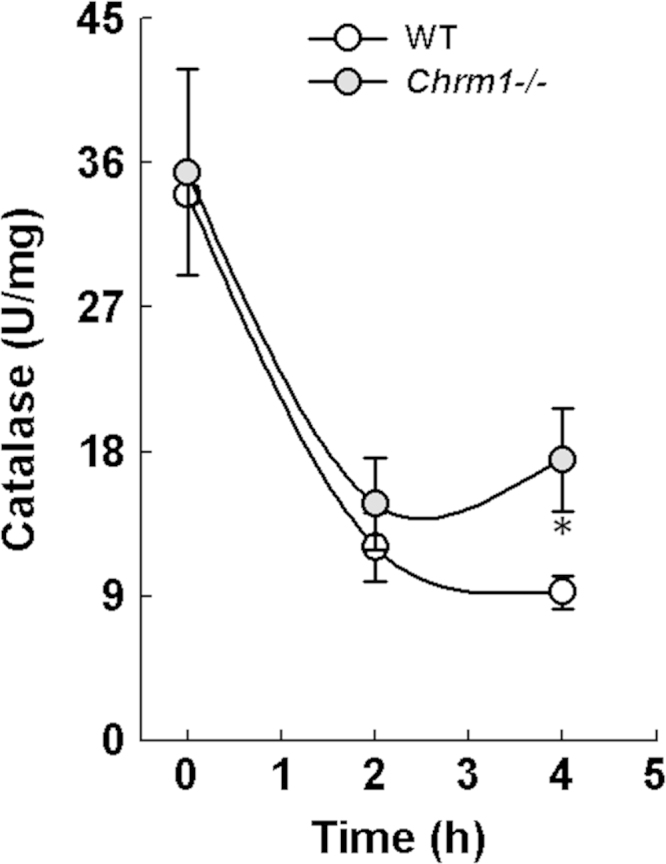
Effect of M1R ablation on APAP-induced changes in hepatic catalase activity. WT and *Chrm1−/−* mice were fasted overnight and treated with APAP 200 mg/kg intraperitoneally in the morning. Two and 4 h after APAP injection, mice were euthanized and their livers harvested and analyzed as described previously [1]. By 2 h, hepatic catalase activity reduced significantly in all APAP-treated mice. However, by 4 h, catalase activity was significantly higher in the livers of *Chrm1−/−* mice when compared to WT mice. Results are mean±S.E.M. * *P*<0.05.

Previously we showed in mice that M1R ablation reduces APAP-induced liver injury by preventing GSH depletion and peroxynitrite generation, thereby reducing hepatocyte necrosis [1]. We also showed that in AML12 hepatocytes (the non-transformed mouse hepatocytes), VU0255035 an M1R antagonist [3], reduced H_2_O_2_-mediated necrosis, an effect abrogated by inhibition of GCLC, the key GSH generating enzyme. Here, we show that unlike M1R antagonist (VU0255035) [1], the novel M1R agonist (VU0357017) [2] did not alter H_2_O_2_-induced cytotoxicity ( Fig. 1). In hepatocytes incubated with H_2_O_2_, VU0255035 augmented catalase activity but had no effect on SOD activity (Fig. 2). Finally, in mice, 2 h after APAP overdose, hepatic catalase activity decreased; however, after 4 h, compared to wild type (WT) mice, catalase activity improved significantly in the livers of M1R-deficient (*Chrm1−/−*) mice (Fig. 3).

## 2 Experimental design, materials and methods

### 2.1 Experimental animals

Animal experiments were conducted in accordance with the *Guide for the Care and Use of Laboratory Animals* prepared by the United States National Academy of Sciences (National Institutes of Health), approved by the Institutional Animal Care and Use Committee, and described in detail previously [1]. The stored liver tissue from the *Chrm1−/−* and WT mice were used to assess catalase activity.

### 2.2 AML12 cell culture

AML12, a non-tumorigenic mouse hepatocyte cell line (ATCC, USA), was cultured at 37 °C with 5% CO_2_ in a 1:1 mixture of Dulbecco׳s modified Eagle׳s and Ham׳s F12 medium with 0.005 mg/ml insulin, 0.005 mg/ml transferrin, 5 ng/ml selenium, and 10% fetal bovine serum. Cells were sub-cultured (1:4 to 1:6) using a 0.25% (w/v) trypsin-0.53 mM EDTA solution.

### 2.3 Cytotoxicity assay

AML12 cells (6.0×10^3^ cells/well) were cultured in 96-well plates in the presence of 1 mM H_2_O_2_ plus vehicle (DMSO) or VU0357017 (0.3–3 μM) (PubChem CID: 25010775), the M1R agonist (Tocris, USA). After 6 h, the culture media was replaced with 100 μl MTT (0.5 mg/ml in culture media) and incubated at 37 °C. After 2.5 h, the MTT solution was discarded, all wells washed with PBS, and added 150 μl DMSO each. The plate was kept at room temperature for 30 min with constant shaking, and absorbance read at 540 nm (VersaMax Microplate Reader, Molecular Device).

### 2.4 Preparation of cell extracts

AML12 cells (1×10^5^ cells/well) were maintained in six-well plates in the presence of 1 mM H_2_O_2_ and vehicle or 1 µM VU0255035 (PubChem CID: 24768606). After 2 h, cells were lysed using a polytron homogenizer using isolation buffer (250 mM sucrose, 10 mM Tris HCL pH 7.4, and 0.1 mM EGTA).

### 2.5 Superoxide dismutase activity assay

SOD activity was estimated as described previously [4]. Reaction mixture contained 0.1 ml phenazine methosulphate (186 μM), 1.2 ml sodium pyrophosphate buffer (0.052 mM, pH 7.0) and 0.3 ml cell supernatant. Enzyme reaction was initiated by adding 0.2 ml NADH (780 μM) and terminated after 1 min by adding 1 ml glacial acetic acid. The chromogen formed was assessed by measuring absorbance at 560 nm on BioMate 3S Spectrophotometer (Thermo Scientific, USA). Results are expressed in units/mg protein.

### 2.6 Catalase activity assay

Catalase activity was assessed in cellular and tissue fractions as described earlier [5]. Briefly, the activity was determined by measuring the decrease in absorbance at 240 nm of a reaction mixture consisting H_2_O_2_ in phosphate buffer, pH 7.0, and cellular/tissue extracts. The molar extinction coefficient of 43.6 M cm^−1^ was used to determine catalase activity and expressed as units/mg protein.

### 2.7 Statistical analysis

All data are expressed as mean±S.E.M. Normality was determined using the Shapiro–Wilk test. Student׳s *t*-test (normally distributed data) or the Mann–Whitney U test (nonparametric data) was used to determine significance. Analysis was performed using SigmaPlot (version 12.0; Systat Software, Inc. San Jose, CA). Significance was defined as *P*<0.05.
